# The *Plasmodium falciparum* Artemisinin Susceptibility-Associated AP-2 Adaptin μ Subunit is Clathrin Independent and Essential for Schizont Maturation

**DOI:** 10.1128/mBio.02918-19

**Published:** 2020-02-25

**Authors:** Ryan C. Henrici, Rachel L. Edwards, Martin Zoltner, Donelly A. van Schalkwyk, Melissa N. Hart, Franziska Mohring, Robert W. Moon, Stephanie D. Nofal, Avnish Patel, Christian Flueck, David A. Baker, Audrey R. Odom John, Mark C. Field, Colin J. Sutherland

**Affiliations:** a Department of Infection Biology, Faculty of Infectious Diseases, London School of Hygiene and Tropical Medicine, London, United Kingdom; b Department of Pediatrics, Washington University School of Medicine, St. Louis, Missouri, USA; c School of Life Sciences, University of Dundee, Dundee, United Kingdom; d Department of Crystallography, Birkbeck, University of London, London, United Kingdom; e Department of Pathogen Molecular Biology, Faculty of Infectious Diseases, London School of Hygiene and Tropical Medicine, London, United Kingdom; f Department of Molecular Microbiology, Washington University School of Medicine, St. Louis, Missouri, USA; g Biology Centre, Institute of Parasitology, Czech Academy of Sciences, Budweis, Czech Republic; h PHE Malaria Reference Laboratory, London School of Hygiene and Tropical Medicine, London, United Kingdom; University of Melbourne; National Institutes of Health

**Keywords:** *Plasmodium falciparum*, adaptin trafficking complex, artemisinin susceptibility, adaptor proteins, endocytosis, malaria

## Abstract

The efficacy of current antimalarial drugs is threatened by reduced susceptibility of Plasmodium falciparum to artemisinin, associated with mutations in *pfkelch13*. Another gene with variants known to modulate the response to artemisinin encodes the μ subunit of the AP-2 adaptin trafficking complex. To elucidate the cellular role of AP-2μ in P. falciparum, we performed a conditional gene knockout, which severely disrupted schizont organization and maturation, leading to mislocalization of key merozoite proteins. AP-2μ is thus essential for blood-stage replication. We generated transgenic P. falciparum parasites expressing hemagglutinin-tagged AP-2μ and examined cellular localization by fluorescence and electron microscopy. Together with mass spectrometry analysis of coimmunoprecipitating proteins, these studies identified AP-2μ-interacting partners, including other AP-2 subunits, the K10 kelch-domain protein, and PfEHD, an effector of endocytosis and lipid mobilization, but no evidence was found of interaction with clathrin, the expected coat protein for AP-2 vesicles. In reverse immunoprecipitation experiments with a clathrin nanobody, other heterotetrameric AP-complexes were shown to interact with clathrin, but AP-2 complex subunits were absent.

## Abstract (precis)

We examine in detail the AP-2 adaptin complex from the malaria parasite Plasmodium falciparum. In most studied organisms, AP-2 is involved in bringing material into the cell from outside, a process called endocytosis. Previous work shows that changes to the μ subunit of AP-2 can contribute to drug resistance. Our experiments show that AP-2 is essential for parasite development in blood but does not have any role in clathrin-mediated endocytosis. This suggests that a specialized function for AP-2 has developed in malaria parasites, and this may be important for understanding its impact on drug resistance.

## INTRODUCTION

Despite important improvements in intervention tools, malaria remains a significant cause of infection and death worldwide, particularly in sub-Saharan Africa. Antimalarial drugs are indispensable components of malaria control, but historical and emerging trends in parasite drug resistance threaten control strategies (1). In uncomplicated Plasmodium falciparum infections, clinical treatment failure following artemisinin combination therapy (ACT) now occurs throughout the Greater Mekong subregion (2–6), with some evidence of decreasing ACT effectiveness in Africa (7–11).

The activity of artemisinin has been linked to parasite hemoglobin metabolism. Heme-derived iron is believed to activate the artemisinin endoperoxide bridge (12), producing oxygen radicals (13). Treatment with protease inhibitors or disruption of falcipain proteases that metabolize hemoglobin reduce parasite susceptibility to artemisinin (14). Mutations in the food vacuole (FV) membrane chloroquine resistance transporter (CRT) reduce susceptibility to chloroquine and piperaquine (15–19), and lineages harboring additional copies of the gene encoding plasmepsin II, another FV protease, have reduced susceptibility to piperaquine (20, 21). However, despite the importance to drug action and intraerythrocytic parasite growth, the mechanisms of uptake of host hemoglobin and transport to the FV are poorly understood. Electron microscopy (EM) studies suggest that hemoglobin enters the asexual parasite through various endocytic events and that hemoglobin is first taken up into small vesicles that form the FV (22, 23). Uptake of host cell components by parasite cytosolic compartments involves some bound by membranes containing phosphoinositide 3-phosphate (PI3P) (24), a marker of endosomal membranes implicated in P. falciparum artemisinin resistance attributed to variants in the propeller region of the kelch domain protein K13 (24–26). Recently, K13 has itself been localized by green fluorescent protein (GFP) tagging to cytoplasmic and peripheral foci in close proximity to the parasite’s FV (27), which may represent the parasite cytostome (28).

In eukaryotes, the process of substrate-specific endocytosis involves clathrin-coated vesicles typically assembled with the adaptor protein 2 (AP-2) complex, a heterotetramer that mediates cargo selection and recruits clathrin to form a coat (29–32). Adaptin functions, which are supported by up to six distinct complexes in some eukaryotes, and clathrin-mediated trafficking have not been analyzed in detail in apicomplexans (29, 33). Although AP-2 has never been studied, the clathrin heavy chain has been localized to post-Golgi secretory structures in the related organism Toxoplasma gondii; some of these are also positive for the AP-1 adaptor complex (34–37). Although no endocytic role in apicomplexan trafficking has been elucidated for clathrin to date, *a priori* AP-2 remains a likely partner of clathrin in P. falciparum.

Our recent data indicate that P. falciparum expressing variants of the μ subunit of AP-2 display reduced susceptibility to artemisinin *in vivo* and *in vitro* (38–40), potentially linking endocytosis to resistance. Given the importance of hemoglobin uptake to parasite survival and drug action in *Plasmodium*, the endocytic function of AP-2 across taxa, and the confirmed role of AP-2 in artemisinin susceptibility, we sought evidence that AP-2μ contributes to clathrin-mediated hemoglobin uptake during asexual parasite development *in vitro*.

## RESULTS

### AP-2μ is localized in the cytosol near the FV and plasma membrane.

**FIG 1:**
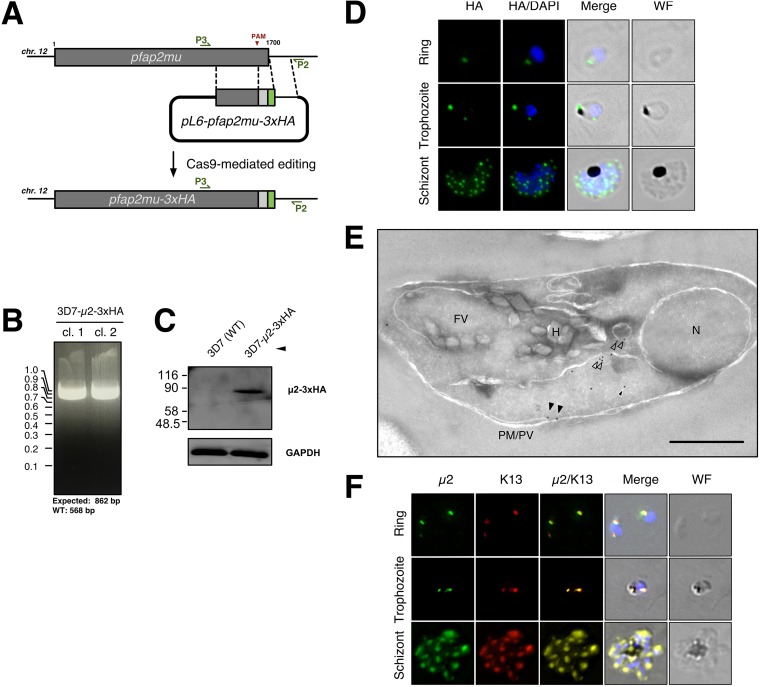
P. falciparum AP-2μ is localized to a noncanonical cytoplasmic compartment. (A) Homologous repair construct used to install AP-2μ variants to fuse a tandem triple hemagglutinin tag (3xHA) onto the C terminus of AP-2μ. (B) PCR-based genotyping of two parasite clones harboring AP-2μ-3xHA in place of the endogenous AP-2μ allele. Amplification of the integrated transgenic *pfap2μ* locus with P3 and P2 (annealing sites annotated) produces an 862-bp fragment. Genotypes were confirmed by Sanger sequencing of the PCR products shown. (C) Anti-HA Western blot confirming expression of the desired fusion protein (∼78 kDa) in mixed-stage lysates, compared to wild-type parental 3D7. Molecular weights are presented in kDa. (D) Localization of AP-2μ-3xHA (green) across the asexual life cycle by anti-HA IFA, counterstained for parasite DNA with DAPI (blue). The images shown are representative of more than 100 cells examined at each stage; merge is the superimposition of each channel on a brightfield image (WF). Maximum intensity z-projections are shown. Scale bar, 2 μm. (E) Immunoelectron micrograph of a representative young intraerythrocytic trophozoite. AP-2μ-3xHA parasites probed with an anti-HA rabbit antibody and a secondary antibody 18 nm gold conjugate. Protein disulfide isomerase (PDI), a marker for the parasite ER, is detected by an anti-PDI mouse antibody and a secondary conjugated to 12-nm gold particles (Fig. S2, S3, S4, and Table S1). N, nucleus; FV, food vacuole; H, hemazoin; PM/PV, plasma membrane/parasitophorous vacuole; empty arrows, AP-2μ associated with vesicles; black arrows, AP-2μ at the plasma membrane; white-outlined arrows, AP-2μ in the cytosol. Scale bar, 500 nm. (F) Localization of AP-2μ-3xHA (green) with respect to episomally expressed GFP-K13 (red) across the asexual life cycle by IFA. Representative images of more than 100 observed cells is shown. Maximum intensity z-projections are shown. Scale bar, 2 μm.

Based on conservation of endocytic machinery across eukaryotic taxa, we expected that AP-2μ would be localized to the parasite periphery where uptake of host cytosol and hemoglobin would occur. A C-terminal triple hemagglutinin (3xHA) epitope tag was introduced by Cas9 editing (Fig. 1A to C) and the localization of AP-2μ-3xHA examined by immunofluorescence assay (IFA) across the asexual blood stages in two independent clones (AP-2μ-3xHA_c1 and AP-2μ-3xHA_c2; Fig. 1B). AP-2μ-3xHA distribution was comparable in both clones, so only data from the first clone are presented.

10.1128/mBio.02918-19.9 TABLE S1 Quantitation of distribution of gold labels in immunoelectron micrographs. Micrographic images of 66 erythrocytes infected with trophozoite-stage parasites expressing 3xHA-tagged AP-2μ, stained with gold particles conjugated to anti-HA antibodies, were examined. For comparison, a similar number of cells infected with trophozoites expressing AP-2μ-2xFKBP-GFP (32), stained with gold particles conjugated to anti-GFP antibodies, were examined. Download Table S1, DOCX file, 0.03 MB. Copyright © 2020 Henrici et al. 2020 Henrici et al. This content is distributed under the terms of the Creative Commons Attribution 4.0 International license.

Throughout the asexual cycle, we observed AP-2μ-3xHA localizing to punctate structures in the parasite cytoplasm (Fig. 1D). In ring-stage trophozoites, AP-2μ appeared as a single cytosolic focus. These foci increased in number as development proceeded and also localized to a cytoplasmic compartment adjacent to the FV in older trophozoites (Fig. 1D, middle row). AP-2μ never labeled the FV membrane. During schizogony, the AP-2μ-labeled structures appeared to replicate and segment into individual daughter merozoites (Fig. 1D, third row).

The cellular distribution of AP-2μ was examined further with respect to a panel of representative organelle markers. The distribution of AP-2μ signal did not overlap with the endoplasmic reticulum (ER), Golgi, or apicoplast markers during development or with the apical secretory organelles during schizogony (see Fig. S1 in the supplemental material).

10.1128/mBio.02918-19.1 FIG S1 Localization of AP-2μ-3xHA with respect to cellular landmarks. Immunofluorescent micrographs of colocalization of AP-2μ-3xHA with several markers of cellular organelles and landmarks in ring stages, trophozoites, and schizonts. Download FIG S1, TIF file, 2.2 MB. Copyright © 2020 Henrici et al. 2020 Henrici et al. This content is distributed under the terms of the Creative Commons Attribution 4.0 International license.

10.1128/mBio.02918-19.2 FIG S2 Localization of AP-2μ-3xHA and Rab5B by immunoelectron microscopy. Electron micrographs of vesicular colocalization of AP-2μ-3xHA (18 nm gold particles) and Rab5B (12 nm gold particles) in developing trophozoites. N, nucleus; ER, endoplasmic reticulum; H, hemozoin; black arrowhead, vesicles colabeled for both proteins. Download FIG S2, TIF file, 1.0 MB. Copyright © 2020 Henrici et al. 2020 Henrici et al. This content is distributed under the terms of the Creative Commons Attribution 4.0 International license.

10.1128/mBio.02918-19.3 FIG S3 Localization of AP-2μ-2xFKBPP-GFP and ER-marker PDI by immunoelectron microscopy. Electron micrograph of typical vesicular colocalization of AP-2μ-2xFKBP-GFP (18 nm gold particles) and PDI (12 nm gold particles) in developing trophozoites. N, nucleus; ER, endoplasmic reticulum; FV, food vacuole; H, hemozoin; black arrow, AP-2μ at the plasma membrane; empty arrow, AP-2μ in vesicles; white-outlined arrows, AP-2μ in the cytosol; white arrows, AP-2μ at the ER. Download FIG S3, TIF file, 2.3 MB. Copyright © 2020 Henrici et al. 2020 Henrici et al. This content is distributed under the terms of the Creative Commons Attribution 4.0 International license.

10.1128/mBio.02918-19.4 FIG S4 Impact of BFA on localization of AP-2μ-3xHA. Treatment of synchronized ring-stage parasite cultures with 5 μg/ml BFA or equivalent methanol solvent for 16 h and immune-stained for AP-2μ (false-colored green) and plasmepsin V (PMV; red). Cells were fixed and stained in suspension and mounted onto coverslips. Pearson’s correlation coefficients (PCC) between indirect AP-2μ and PMV signals were calculated using Nikon AR Analysis software. The mean of at least 20 cells with standard deviation is shown. PCC were significantly different (***, *P* < 0.005) using Student’s *t* test. Download FIG S4, TIF file, 0.3 MB. Copyright © 2020 Henrici et al. 2020 Henrici et al. This content is distributed under the terms of the Creative Commons Attribution 4.0 International license.

To better characterize the localization of AP-2μ, we performed immunoelectron microscopy (immuno-EM) on thin sections of trophozoites expressing AP-2μ-3xHA. In an analysis of 66 micrographs of single parasite-infected erythrocytes, gold particles detecting anti-hemagglutinin (anti-HA) antibodies bound to AP-2μ-3xHA were observed near the ER (73.8% of micrographs), in vesicles in the cytosol (37.9%), in tubular cytosolic structures (93.6%), near the FV (5.8%), and at the parasite plasma membrane (4.2%) (Fig. 1E; Table S1). At least some cytosolic AP-2μ-positive vesicles also contained Rab5B, an effector of endosome-like transport between the plasma membrane and the FV (Fig. S2) (41). Parasites expressing AP-2μ-2xFKBP-GFP showed a similar localization and distribution by immuno-EM (Fig. S3; Table S1), but GFP fluorescence was too faint to reliably observe in live cells.

*Plasmodium* parasites lack a stacked Golgi apparatus, and differentiating the ER from the Golgi apparatus by EM is difficult. Therefore, AP-2μ-3xHA parasites were treated with brefeldin A (BFA), a fungal metabolite and fast-acting inhibitor of ER-to-Golgi secretory traffic. Upon stimulation with BFA, proteins localized to, or trafficked via, the Golgi compartment relocalize to the ER. Previous studies examining intracellular traffic in *Plasmodium* have shown that parasite cultures remain viable when treated with 5 μg/ml BFA for up to 24 h (42). After treating ring-stage parasites with 5 μg/ml BFA for 16 h, AP-2μ-3xHA staining significantly colocalized with staining observed for plasmepsin V, a luminal ER protease, suggesting AP-2μ is localized to, or via, a secretory membrane. AP-2μ and plasmepsin V staining were distinct in solvent-treated controls (see Fig. S4 in the supplemental material).

In recent studies, K13 has been localized to conspicuous membranous structures in the cytosol near the FV (27) or plasma membrane (28), and these superficially resemble structures labeled by AP-2μ here (Fig. 1D and E). Given the apparent similarity in cellular distribution and importance of both molecules in ring-stage artemisinin susceptibility *in vitro*, we hypothesized that AP-2μ and K13 localize to the same cytosolic compartment. We overexpressed an episomally encoded N-terminal GFP-K13 fusion protein in our AP-2μ-3xHA-expressing line and observed GFP-K13 signal resembling that of previous studies (27). K13 and AP-2μ displayed a striking similarity in signal distribution in ring and schizont stages (Fig. 1F).

### PfAP-2μ is required for asexual replication.

**FIG 2:**
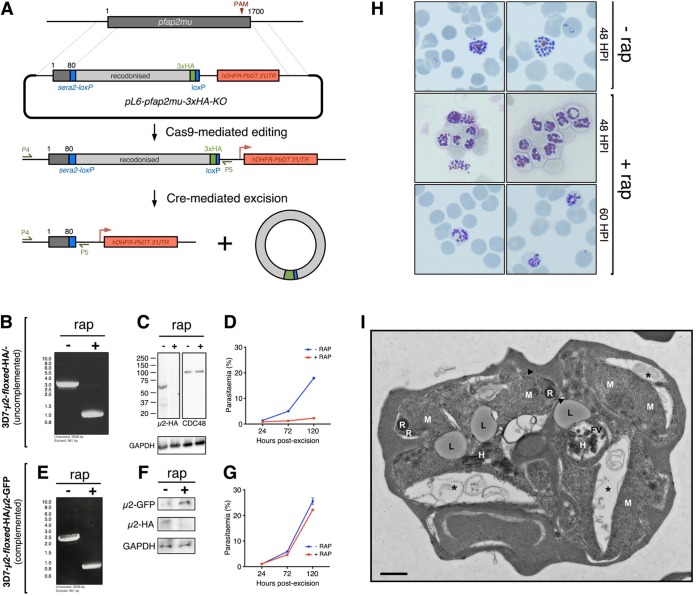
AP-2μ is required for asexual replication and schizont maturation. (A) Schematic for the integration of *loxP* recombination elements into the endogenous *pfap2μ* locus of a parasite line constitutively expressing a split-Cre recombinase (43). The addition of rap initiates Cre dimerization and excision of the *loxP*-flanked (*floxed*) region of *pfap2μ* on chromosome 12. (B) PCR confirmation of rap-induced excision of *floxed* region by PCR using the primers P4 and P5 (see panel A). (C) Western blot confirmation that excision of *floxed pfap2μ* causes a loss of AP-2μ-3xHA protein (within 24 h) but has no effect on levels of CDC48 protein. Molecular weight is presented in kDa. (D) Parasite multiplication in the 3D7-AP-2μ-*floxed*-3xHA line across 2.5 cell cycles, with or without rap induction of Cre. The mean parasitemia (normalized to 0.25% starting parasitemia) with the standard error is shown at each time point. Each data point represents the average of at least three biological replicates (different cultures, different days). (E) PCR confirmation of rap-mediated *pfap2μ* excision in 3D7-AP-2μ-*floxed*-3xHA parasites transfected with an episome encoding *cam-*AP-2μ-GFP. The construction of this complementation plasmid is described in Fig. S5. (F) Western blot confirmation that excision of chromosomal *pfap2μ* from 3D7-AP-2μ-*floxed*-3xHA/*cam-*AP-2μ-GFP parasites causes a loss of AP-2μ-3xHA protein, but it does not prevent episomal expression of AP-2μ-GFP. (G) Parasite multiplication in the 3D7-AP-2μ-*floxed*-3xHA/*cam-*AP-2μ-GFP line across 2.5 cell cycles, with or without induction of Cre by rap. Means and standard errors are shown as in panel D. (H) Giemsa staining of 3D7-AP-2μ-*floxed*-3xHA schizonts, without rap treatment at 48 h postinfection and with rap treatment at 48 and 60 h postinfection. (I) Electron micrograph of 3D7-AP-2μ-*floxed*-3xHA schizonts, with or without 1-h ring-stage treatment with 10 nM rap. Micronemes at the apical end of developing merozoites are labeled with arrowheads; asterisks indicate membrane separation (see Fig. S7). FV, food (digestive) vacuole; H, hemozoin; L, lipid body; M, merozoite; R, rhoptry. Scale bar, 500 nm.

Given its proximity and localization at the plasma membrane and to cytosolic vesicles, our data suggest a relationship between AP-2 and the FV. However, the apparent presence of AP-2 in secretory ER structures by EM appears to conflict with this model. To better characterize the role of AP-2, we deployed an inducible DiCre system to study the impact of conditional *pfap2μ* knockout (KO) *in vitro* (43). Specifically, the Cas9 donor constructs described above were modified to insert both a *loxP* site into the 3′ untranslated region (UTR) immediately after the *pfap2μ* stop codon and a *loxP-*containing *pfsera2* intron into the 5′ end of the gene, 261 bp downstream of the translation start, such that Cre-mediated excision removes the majority of the coding sequence, including the 3xHA tag. These constructs were introduced into 3D7 parasites constitutively expressing dimerizable Cre recombinase (44) (Fig. 2A).

10.1128/mBio.02918-19.5 FIG S5 Schematic of episomal complementation of AP-2μ conditional knockout. The *floxed* AP-2μ-3xHA- and DiCre-expressing parasite line 3D7-μ2-floxed-3xHA was transfected with a plasmid that constitutively expresses μ2-GFP under the *cam* promoter (pDC2-*cam-pfap2μ-GFP-BSD*). These cells were maintained on 2.5 μg/ml blasticidin S (BSD). Upon addition of rap, split Cre recombinase dimerizes and excises the endogenous *pfap2μ* locus on chromosome 12. Parasites still express μ2-GFP via the pDC2 episome. Download FIG S5, TIF file, 0.4 MB. Copyright © 2020 Henrici et al. 2020 Henrici et al. This content is distributed under the terms of the Creative Commons Attribution 4.0 International license.

Ring-stage cultures of the 3D7-AP-2μ-*floxed*-3xHA parasites were treated with 10 nM rapamycin (rap) for 30 min to dimerize the split Cre recombinase and trigger *pfap2μ* excision. Genomic DNA was extracted 24 h after this treatment, and PCR confirmed complete excision of the *floxed* region of *pfap2μ* (Fig. 2B), resulting in ablation of AP-2μ protein expression (Fig. 2C). Parasite counts by fluorescence-activated cell sorting (FACS) revealed that induced-KO of *pfap2μ* prevented parasite replication within a single asexual cycle, without appreciable recovery over multiple cycles (Fig. 2D), showing that *pfap2μ* is required for asexual replication *in vitro*. Importantly, the Cre-mediated endogenous *pfap2μ* KO was fully complemented with an episomally expressed copy of AP-2μ-GFP under a constitutive promoter (Fig. 2E to G; Fig. S5). The finding that disruption of the μ subunit is lethal confirms that integration of the C-terminal tandem HA tag on the μ subunit does not significantly disrupt the AP-2 complex, as our tagged parasites grew normally.

When examined by Giemsa staining, parasites lacking *pfap2μ* arrest as malformed schizonts which are still present in the culture at 60 h postinvasion (Fig. 2H). These defective schizonts occupy approximately half of the red cell cytoplasm and contain poorly segmented merozoites compared to wild-type schizonts. Consistent with reduced size, *pfap2μ* KO schizonts carried fewer nuclei (mean ± standard deviation [KO, 13.0 ± 4.9; wild type, 19.3 ± 4.8; *P* < 0.0001; *n* = 50 KO and 50 wild type]). As determined by FACS, the mean DNA content may be slightly lower in KO schizonts (mean ± standard deviation [KO, 18,180 ± 8,900 U; wild type, 19,980 ± 10,000 U; *P* < 0.001; *n* = 21,924 events [KO] and 21,552 events [wild type]), but this difference is small and does not explain the more than 45% difference in number of segmented nuclei (Fig. S6). Rarely, rap-treated parasites were observed to undergo egress and invasion, probably due to occasional failure to excise *pfap2μ*.

10.1128/mBio.02918-19.6 FIG S6 AP-2μ-KO schizonts have similar nucleic acid content to wild-type schizonts but fewer discrete nuclei. (A) Swarm plot depicting number of nuclei per schizont. 50 schizonts were counted for AP-2μ wild-type (left) and knockout (right) schizonts. Overlaying box plot show median and IQR. ***, *P* < 0.0001 for a Mann-Whitney comparison of medians. (B) Representative histogram comparing DNA/RNA content in wild-type (-rap, blue) and AP-2μ-KO (+rap, red) schizonts. Nucleic acid was stained using SYBR green, and SYBR green signal was detected in live cells by FACS. Rap– parasites were egress blocked by treatment with the reversible PKG inhibitor compound 2. Download FIG S6, TIF file, 2.1 MB. Copyright © 2020 Henrici et al. 2020 Henrici et al. This content is distributed under the terms of the Creative Commons Attribution 4.0 International license.

10.1128/mBio.02918-19.7 FIG S7 Impact of AP-2μ KO on trophozoite and schizont maturation and morphology. Electron micrographs examining the morphology of wild-type (-rap) and AP-2μ-KO (+rap) trophozoites (top panel) and schizonts (bottom panel). Two representative micrographs are shown for each cell type. Rap or equivalent DMSO was added to synchronous ring-stage 3D7-AP-2μ-*floxed*-3xHA parasites for 1 h, and cells were fixed for imaging in the same development cycle. Labels used in trophozoite micrographs N, nucleus; FV, food (digestive) vacuole; C, cytostome; A, apicoplast. A total of 300 schizonts from each treatment were systematically enumerated for key features (see main text). Black arrowheads indicate accumulated lipid bodies in AP-2μ-KO schizonts. Download FIG S7, TIF file, 1.3 MB. Copyright © 2020 Henrici et al. 2020 Henrici et al. This content is distributed under the terms of the Creative Commons Attribution 4.0 International license.

### PfAP-2μ is required for schizogony.

Examining malformed AP-2μ-KO schizonts by electron microscopy revealed gross morphological defects during schizogony and merozoite biogenesis (Fig. 2I; Fig. S7). Merozoites forming within these schizonts are highly disorganized and misshapen within the parasitophorous vacuole membrane, and there are large pockets of schizont cytosol between the malformed merozoites. The membranes seem indistinct, loosely encircling the deformed merozoites with irregular invaginations not seen in wild-type parasites (Fig. 2I; Fig. S7), and were consistently poorly preserved during fixation and processing for electron microscopy despite several replicate preparations. These bilayers tended to separate dramatically compared to membranes in wild-type schizonts, and we cautiously attribute this observation to membrane fragility in the absence of AP-2μ.

In addition, electron micrographs revealed a statistically significant accumulation of lipid bodies in the cytosol of AP-2μ KO schizonts (Fig. 2I; Fig. S7), with some cells having two, three, or four such bodies (Poisson regression: coefficient, 0.597; 95% confidence interval [CI] = 0.344 to 0.850; *P* < 0.001; *n* = 300 treated and 300 untreated). These parasites were also more likely to contain aberrant FV that appeared fragmented or elongated (odds ratio, 3.18; 95% CI = 1.89 to 5.48; *P* < 0.0001). Consistent with this, apparently free hemozoin crystals were occasionally observed in the schizont cytosol. Wild-type and KO trophozoites appear to be morphologically equivalent (Fig. S7), and apicoplasts can be observed in these cells.

**FIG 3:**
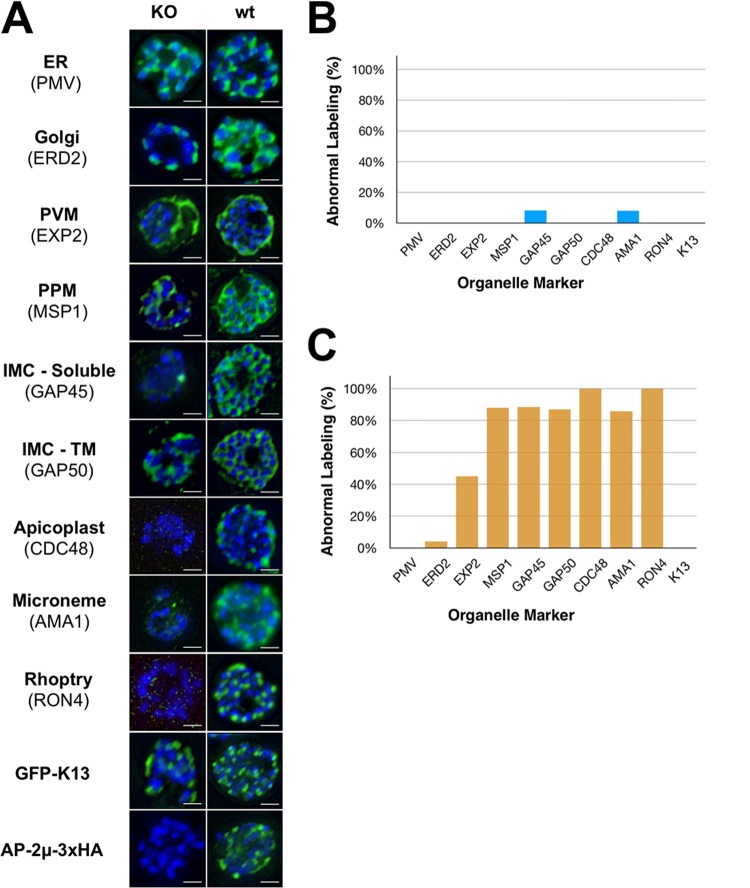
AP-2μ-KO severely disrupts schizont maturation. (A) Antibodies against the ER (PMV), Golgi apparatus (ERD2), PVM (EXP2), PPM (MSP1), IMC (GAP45, GAP50), apicoplast (CDC48), micronemes (AMA1), rhoptries (RON4), episomal K13 (GFP), and AP-2μ (HA) were used to stain 3D7-AP-2μ-*floxed*-3xHA schizonts with rap treatment (KO) or without (wt). All organelle markers have been false colored green regardless of fluorophore-conjugated secondary antibody used for clarity. Nuclei have been false colored blue. IMC-TM, transmembrane component of inner membrane complex. Scale bar, 2 μm. (B and C) Abnormal labeling in AP-2μ KO parasites was quantitated relative to the staining observed in the majority of wild-type schizonts (B rap–; C rap+). Normal staining was defined as follows: ERD2, PMV, and CDC48, discrete punctate staining corresponding to each nucleus; EXP2, contiguous, circular, peripheral membrane staining; GAP45, GAP50, and MSP1, distinct, circular grape-like staining surrounding each daughter nucleus; AMA1 and RON4, discrete, apical punctate spots corresponding to each nucleus. At least 100 cells were scored for each marker.

Subsequent IFA imaging supported these findings, since deletion of *pfap2μ* disrupts the biogenesis of several membrane-bound organelles (Fig. 3). Specifically, AMA1, RON4, and CDC48, markers of the micronemes, rhoptries, and apicoplast, respectively, are mislocalized by IFA, despite being detectable at normal levels by Western blotting (Fig. S8). Despite this, rhoptries and micronemes are visible in some KO schizont EM sections (Fig. 2I; Fig. S7), implying a defect in transport rather than organelle biogenesis. In addition, in KO schizonts, MSP1 antibody staining is indistinct and fails to delineate nascent merozoites, suggesting that invagination of the plasma membrane during schizogony may be disrupted in the absence of AP-2μ (Fig. 3). The parasitophorous vacuole membrane, labeled by EXP2, seems to be largely intact though may have discontinuities in some cells. The ER and *cis-*Golgi compartment display no obvious abnormalities in cells lacking AP-2μ (Fig. 3). Overall, it is unlikely that these widespread defects are all directly attributable to AP-2μ, but rather that this complex phenotype arises from knock-on effects of AP-2μ deletion affecting downstream effector molecules.

10.1128/mBio.02918-19.8 FIG S8 Organelle markers are mislocalized by AP-2μ KO. Antibodies against RON4, AMA1, and CDC48, which had previously failed to stain schizonts lacking AP-2μ (Fig. 3), were deployed in Western blot analyses of whole-cell lysates prepared from wild-type and AP-2μ knockout schizonts. The overall cellular abundance of these factors was not affected by AP-2μ knockout. Download FIG S8, TIF file, 0.10 MB. Copyright © 2020 Henrici et al. 2020 Henrici et al. This content is distributed under the terms of the Creative Commons Attribution 4.0 International license.

### The AP-2 complex is clathrin independent and associates with Kelch10.

To identify AP-2μ interacting partners, early schizont (32 to 36 h postinvasion) cell lysates of P. falciparum AP-2μ-3xHA were prepared using cryomilling and detergent lysis, a cell disruption technique that has generated high-resolution interactomes in other organisms, although not previously deployed in *Plasmodium* (45). Using both Triton X-100 and CHAPS-containing lysis buffers, originally derived for the extraction of clathrin-interacting proteins from *Trypanosoma* species, we lysed the frozen parasites, immunoprecipitated AP-2μ using anti-HA-conjugated beads and performed mass spectrometry (MS).

**FIG 4:**
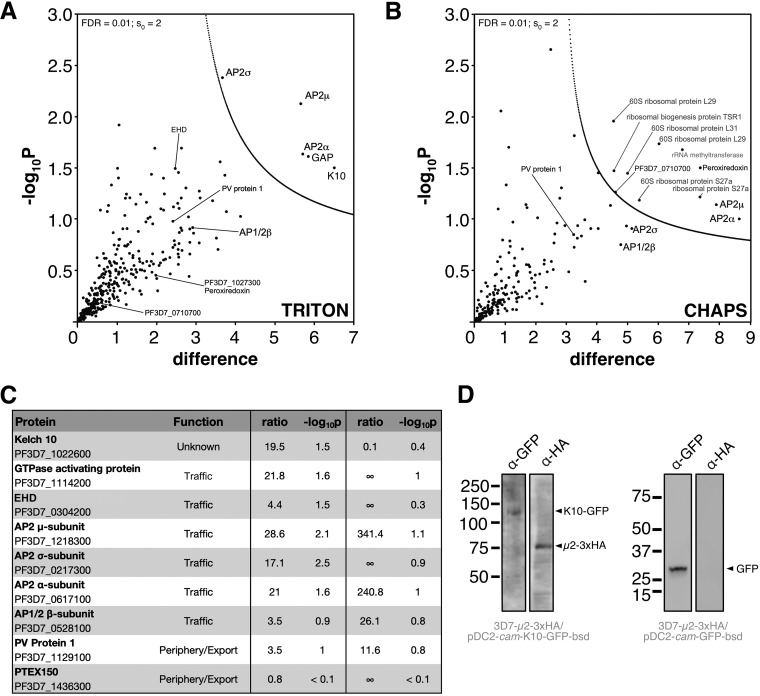
Identification of AP-2μ-interacting proteins. (A) Volcano plot from *P* values versus the corresponding *t* test difference of proteins identified by immunoprecipitation (IP) in Triton buffer. Cutoff curves for statistically significant interactors (dotted curve) were calculated from the estimated false discovery rate (for details, see Materials and Methods). Selected hits are labeled (potentially nonspecific interactors are in gray). (B) Volcano plot for proteins identified by IP in CHAPS buffer. (C) Table of selected identified interactors listing functional annotation, enrichment ratios (compared to controls; see Materials and Methods) and negative log_10_ of corresponding *P* values for Triton and CHAPS buffers, respectively. Additional hits are listed in an extended table available at https://doi.org/10.17037/DATA.00001533. (D) *pfk10-*GFP (left panel) or GFP alone (right panel), driven by the calmodulin promoter, was expressed episomally in 3D7-AP-2μ-3xHA parasites and immunoprecipitated with α-GFP antibody-coated magnetic beads. Western blots of fractionated proteins are shown, probed with either α-GFP or α-HA antibodies. Molecular weight is presented in kDa.

Since AP-2 is the canonical eukaryotic clathrin-interacting endocytic complex, we expected AP-2μ to interact with clathrin and the other AP-2 subunits in P. falciparum. Indeed, all four AP-2 complex subunits were enriched under both lysis conditions tested, demonstrating that the complex in P. falciparum comprises subunits annotated in the genome as AP-2α, AP-2σ, AP-2μ (our tagged bait protein), and AP-1β, previously predicted to be shared between the AP-1 and AP-2 complexes (Fig. 4A to C) (46, 47). The latter subunit is therefore designated AP-1/2β. Putative nucleotide-dependent regulators of vesicular traffic and a kelch-type beta-propeller domain protein encoded on chromosome 10, K10, were also identified with high confidence under one or both lysis conditions (Fig. 4C; https://doi.org/10.17037/DATA.00001533). We confirmed, by Western blotting, that AP-2μ-3xHA copurifies with episomally expressed and immunoprecipitated K10-GFP but not similarly expressed cytosolic GFP (Fig. 4D). Despite little sequence identity between K10 and K13, a codon 623 polymorphism in the locus encoding K10 has been identified as coselected with K13 variants in artemisinin-resistant parasites (48). K13 was not identified as an AP-2μ-interacting protein in our immunoprecipitations. In addition, PfEHD, previously associated with endocytosis and lipid storage, was enriched. Strikingly, neither the clathrin heavy chain (CHC; gene ID PF3D7_1219100) nor the clathrin light chain (CLC; gene ID PF3D7_1435500) was enriched in either extraction condition with our HA-tagged AP-2μ (https://doi.org/10.17037/DATA.00001533).

**FIG 5:**
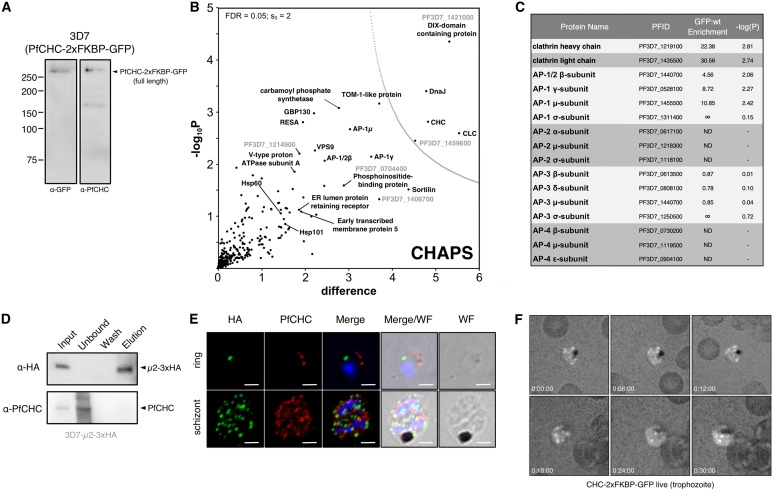
P. falciparum AP-2μ does not interact with clathrin heavy chain. (A) Western blot of mixed-stage 3D7-CHC-2xFKBP-GFP lysates probed with antibodies, either α-GFP (left) or α-PfCHC (right). (B) Volcano plot from *P* values versus the corresponding *t* test difference of proteins identified by α-GFP (nanobody) immunoprecipitation in CHAPS buffer. Cutoff curves for statistically significant interactors (dotted curve) were calculated from the estimated false discovery rate. (C) Abundance/enrichment ratio table for subunits of all adaptin subunits identified in the α-CHC-GFP pulldown. Additional hits are listed in an extended table available at https://doi.org/10.17037/DATA.00001533. ND, no peptides were detected that correspond to the listed protein. (D) Western blot of α-PfCHC immunoprecipitation performed on cryomilled 3D7-AP-2μ-3xHA trophozoite lysates. Membrane was probed with α-PfCHC and α-HA antibodies. (E) Maximum intensity projection IFA of representative trophozoite and schizont stages of 3D7-AP-2μ-3xHA parasites, from among at least 100 cells examined at each stage, probed with both α-HA antibodies and α-PfCHC, which are green and red in the merge images, respectively. Scale bar, 2 μm. (F) Representative maximum intensity projection images of time-lapse live microscopic observation of CHC-2xFKBP-GFP in a trophozoite. Each frame represents the passage of 6 min.

To further validate our observation that AP-2 does not appear to interact with CHC, we performed a similar analysis on a trophozoite preparation of parasites expressing CHC-2xFKBP-GFP (Fig. 5A). All AP-1 complex components were enriched in these PfCHC pulldowns, including AP-1/2β, as were other trafficking-associated components (Fig. 5B and C; https://doi.org/10.17037/DATA.00001533). However, no peptides from AP-2 α, μ, and σ subunits were identified in MS analysis of four replicate pulldowns (Fig. 5B and C). Given the dual presence of AP-1/2β as a component of both AP-1 and AP-2, we consider the lack of additional AP-2 subunits in these immunoprecipitation (IP) experiments to be strong evidence for the absence of an AP-2/CHC interaction. The lack of AP-2 involvement in clathrin-dependent endocytosis has been demonstrated in African trypanosomes, where the genes encoding the AP-2 subunits are absent and also suggested in Trypanosoma cruzi, where clathrin does not appear to interact with AP-2 (45), but has never before been demonstrated in *Plasmodium* (34, 46). AP-3 complex subunits were also identified, but they were not enriched compared to controls. The role of the AP-4 complex is unclear in eukaryotes, but this complex is not believed to interact with clathrin. Consistent with this, we did not detect peptides corresponding to the P. falciparum AP-4 complex. Interestingly, peptides corresponding to Sortilin, Vps9 (a DnaJ chaperone), and ring-stage infected erythrocyte surface antigen (RESA) were enriched in the clathrin interactome, among other exported and trafficking-related factors. Collectively, these data suggest that retromer, *trans*-Golgi, and secretory trafficking likely involve clathrin-coated vesicles (https://doi.org/10.17037/DATA.00001533).

The absence of interacting clathrin was further investigated by direct IP of CHC from P. falciparum AP-2μ-3xHA lysates and Western blotting. We found no evidence that AP-2μ-3xHA copurifies with immunoprecipitated CHC (Fig. 5D). Using an anti-P. falciparum clathrin antibody, validated on a parasite line expressing PfCHC-2xFKBP-GFP (Fig. 5A), we also found evidence by IFA that AP-2μ-3xHA and CHC are localized to separate compartments (Fig. 5E). Consistent with our clathrin interactome, our localization of PfCHC demonstrates many rapidly cycling foci decorating the plasma membrane and cytoplasmic structures in live trophozoites (Fig. 5F). This dynamic localization was never observed for AP-2 in our imaging studies. These data therefore support a novel, clathrin-independent role for the AP-2 complex in *Plasmodium*.

## DISCUSSION

We investigated the location and function of the μ subunit of the AP-2 adaptor complex in P. falciparum as a window into endocytosis, a major mechanism by which the parasite ingests hemoglobin. This process supports parasite metabolism but also provides the target of many frontline antimalarials. Little is known about endocytic mechanisms in *Plasmodium*. Virtually all other eukaryotes exploit a clathrin-based mechanism for sampling the extracellular space and uptake of specific molecules, and we expected P. falciparum to utilize a similar machinery. This first characterization of AP-2 in apicomplexans highlights significant divergence from eukaryotic canonical endocytic mechanisms and previously established roles for AP-2 and clathrin.

Light-level imaging and immuno-EM localized the *Pf*AP-2 complex to the plasma membrane, as well as to distinct cytoplasmic foci, corresponding to vesicles near the FV during intraerythrocytic development. These structures are visible throughout the asexual cycle and replicate and segregate into merozoites during schizogony. By colabeling immuno-EM, we show that a subset of these vesicles are also decorated with Rab5B, a GTPase that regulates traffic at the parasite plasma membrane, endosomes, and digestive vacuole (41). Inhibition of intra-Golgi trafficking with brefeldin A suggests that AP-2 arrives at these locations *via* Golgi compartment-dependent vesicular transport routes.

Importantly, our proteomic experiments defined the AP-2 complex components in P. falciparum as α, μ, and σ subunits plus the β subunit that participates in both AP-1 and AP-2 complexes. Genes encoding β1, β3, and β4 are annotated in the genome, and there appears to be no discrete β2 (46), supporting a dual-purpose β1 in P. falciparum. This promiscuous behavior has been reported previously in other organisms, where β1/2 has functional importance for targeting the vesicular complex to specific membranes (47). Further work is required to determine whether this is also true of β in *Plasmodium*, but it is clear that the protein is a *bona fide* member of both AP-1 and AP-2 complexes. Strikingly, we found no evidence of clathrin heavy or light chains in our AP-2μ interactome or of AP-2 subunits in our CHC interactome, which did include all four components of AP-1 (34). Clathrin was localized to punctate structures throughout the parasite cytosol, a distribution dramatically different to that for AP-2μ, consistent with a lack of interaction (Fig. 5). A similar result was obtained for AP-2 in T. cruzi using comparable techniques, suggesting clathrin and AP-2 may not be universally associated, as previously thought (45). These data imply that despite the shared β subunit, which canonically links the AP complex to clathrin, other unknown P. falciparum subunits or factors may be involved in the selection of coat proteins. These remain unidentified for AP-2 but may lie among the many proteins of unknown function identified in our AP-2 interactome, although no obvious coat scaffold proteins were present (Fig. 4; http://datacompass.lshtm.ac.uk/1461/). Future studies should aim to further define AP-2- and clathrin-mediated traffic in Apicomplexa and establish whether other adaptins perform diverged roles. Clathrin-independent AP-2 trafficking may prove widespread, since its occurrence in two very divergent protists suggests this may be a more common phenomenon.

Although we cannot define exactly the functions of AP-2 in *Plasmodium*, several lines of evidence support a role in clathrin-independent endosomal transport, possibly linked to the membrane recycling pathway. First, we colocalized AP-2 and Rab5B, a mediator of FV and plasma membrane transport in P. falciparum, to discrete vesicles by immuno-EM microscopy. These most likely represent a subset of endosomal structures. Next, conditional deletion of AP-2μ causes profound defects in membrane segregation, lipid accumulation, and FV integrity during schizogony, ultimately causing arrest of intraerythrocytic development. Although we do not observe the accumulation of red blood cell (RBC) cytosol-containing vesicles at the plasma membrane, as observed in “knock-sideways” experiments with the PIP3-linked kinase VPS45 (24), deletion of AP-2μ may block vesicular formation as AP-2 is responsible for selective concentration of cargo into a nascent transport vesicle in most organisms where this has been examined. In addition, parasites lacking AP-2μ occupy less than half of the RBC cytosol, a potential consequence of reduced endocytosis and disrupted growth. Lastly, our proteomic investigation reveals AP-2 interacts with a number of vesicular cofactors, including PfEHD, previously associated with endocytosis and lipid mobilization in P. falciparum.

AP-2μ conditional knockout leads to disruption and mislocalization of a subset of proteins normally trafficked to the apical secretory organelles of nascent merozoites. Such mistargeting of cargo proteins is likely to have pleotrophic impacts as cellular components fail to reach the correct compartment or are present at an inappropriate level. Dissecting this in detail, using for example the knock-sideways strategy (27), will allow more detailed interrogation of stage-specific effects of AP-2μ depletion, as will analysis of hemoglobin trafficking.

Interestingly, though we found no evidence of a direct interaction between them, we did find that structures labeled by AP-2μ overlap structures labeled by K13, the major gene underlying artemisinin susceptibility in Southeast Asia (5). Yang et al. recently demonstrated that K13 is localized to doughnut-shaped peripheral structures resembling a collar of the cytostome, an endocytic invagination of the plasma and parasitophorous vacuole membranes that delivers hemoglobin-rich host cell cytoplasm to the FV (28). These observations are compatible with our results since hemoglobin-filled vesicles, presumably defined by AP-2 based on our results here, bud from the distal cytostome and traffic to the FV by an actin-myosin mechanism, and superresolution methodologies might help resolve this localization and function (49, 50). In other studies, K13 also localizes to PI3P-labeled structures implicated in modulation of artemisinin susceptibility (25, 26). In *Plasmodium*, PI3P is a membrane component of endocytic vesicles (24, 51). Thus, our findings and those of other investigators support a role for endocytosis, hemoglobin ingestion, and more generally intracellular traffic in artemisinin susceptibility. First, K13 is the primary determinant of reduced susceptibility in Southeast Asia and has now been implicated in cytostomal ingestion of host cytoplasm (28). Second, mutations in the trafficking adapter protein AP-2μ are linked to clinical responses to ACT in human infections and parasite artemisinin susceptibility *in vitro* (8, 38–40). Third, mutations in the actin-binding protein Coronin also cause P. falciparum ring-stage artemisinin resistance *in vitro* (52–54). Coronin has been linked to regulation of endocytosis in Toxoplasma gondii (52). In addition, a mutation in AP-2α was recently identified in a laboratory-evolved lineage with reduced susceptibility to artemisinin (55), and K10, an AP-2μ interacting partner, has been implicated in the complex multigenic signature of artemisinin susceptibility in Southeast Asia (48). Further, a recent study showing that AP-2μ and K13 are essential for a clathrin-independent endocytic mechanism of ART resistance found no evidence of a direct interaction between the two (56), which is consistent with our data. However, both studies found PF3D7_081300 (KIC7) in the respective K13 and AP-2μ interactomes, and thus KIC7 may be a functional link between the two factors. These data justify further functional studies of K13, AP-2, Coronin, PI3P, K10, and KIC7 toward a mechanistic understanding of how cellular trafficking components modulate artemisinin susceptibility in P. falciparum.

Here, we show that AP-2μ contributes to the processes of endocytosis and intracellular traffic during parasite development and is essential for intraerythrocytic schizont maturation. Further defining endocytosis in P. falciparum will provide key insights into both the secretory system, which is important for replication, invasion and immune evasion, and drug susceptibility. Our study provides the first comprehensive scrutiny of clathrin and AP-2 functions in *Plasmodium* and identifies divergence from other eukaryotes that may be an important feature of the evolution of parasitism in apicomplexans.

## MATERIALS AND METHODS

### Plasmid design and construction.

Plasmids pL6-AP2μ-3xHA-sgDNA and pL6-AP2μ-*floxed*-3xHA-sgDNA encoding the donor sequences carried the tandem epitope tag and recombination elements were created for transfecting 3D7 parasites as described previously (57). The coding sequence between the epitope tag and upstream synthetic *sera2* intron containing LoxP was recodonized to facilitate efficient homologous recombination (synthesis by Invitrogen) (43). These elements were cloned between two 500-bp sequences homologous to the 5′ UTR and the 3′ UTR of *pfap2μ*.

### Parasite culture and generation of transgenic parasites.

Plasmodium falciparum culture was performed as described previously (40). Two transfection methods were used in this study. For the integration of single nucleotide polymorphisms, ring-stage transfection was performed. Briefly, 3D7 parasites were cultured to approximately 10 to 15% parasitemia in 5% hematocrit under standard conditions. Immediately before transfection, 100 μg of each plasmid (pL6 and pUF1-Cas9) was ethanol-acetate precipitated and resuspended in 100 μl of sterile Tris-EDTA. Next, 300 μl of infected RBC were isolated by centrifugation and equilibrated in 1× Cytomix (120 mM KCl, 5 mM MgCl_2_, 25 mM HEPES, 0.15 mM CaCl_2_, 2 mM EGTA, 10 mM KH_2_PO_4_/K_2_HPO_4_ [pH 7.6]). Then, 250 μl of packed cells was combined with 250 μl of 1× Cytomix in a 2-mm transfection cuvette (Bio-Rad Laboratories). The precipitated and resuspended DNA was added to the cell suspension in the cuvette. The cells were immediately pulsed at 310 V, 950 μF, and infinite resistance in a Bio-Rad Gene Pulser. The electroporated cells were then washed twice in complete medium to remove debris and returned to culture. Fresh red blood cells were added to approximately 5% total hematocrit on day 1 after transfection along with 2.5 nM WR99210 and 1.5 μM DSM-1. Media and selection drugs were replenished every day for 14 days and then every 3 days until parasites were observed by microscopy. Parasites recovered at approximately 3 weeks posttransfection. The tagged μ2 parasite line was created by the spontaneous DNA uptake method exactly as described by Deitsch et al. (58). The 3D7-μ2-2xFKBP-GFP and 3D7-CHC-2xFKBP-GFP parasite lines, generated via selection-linked integration (27), were generously provided by Tobias Spielmann. The 3D7 DiCre-expressing parasite line (43) was generously provided by Michael Blackman.

### Fluorescence microscopy.

Immunofluorescence microscopy was performed on thin smears dried on glass slides, fixed for 10 min with 4% formaldehyde in phosphate-buffered saline (PBS), washed three times with PBS, permeabilized with 0.1% (vol/vol) Triton X-100 in PBS for 10 min, washed again, and blocked for 1 h with 3% (wt/vol) bovine serum albumin (BSA) in PBS. Primary antibodies were diluted in PBS containing 3% (wt/vol) BSA and 0.1% (vol/vol) Tween 20 and then incubated on the slide overnight at 4°C. The slides were again washed several times with PBS, incubated with secondary antibodies diluted in the same buffer, incubated for 1 h at room temperature, and washed. Glass coverslips were mounted with 1 μl of Vectashield with DAPI (4′,6′-diamidino-2-phenylindole). Images were taken on a Nikon TE-100 inverted microscope.

### Electron microscopy.

For ultrastructural analysis of 3D7-AP-2μ-*floxed*-3xHA, parasites were cultured at 37°C in 4-ml volumes in six-well tissue culture dishes (Techno Plastic Products) at 2% hematocrit until reaching 6 to 10% parasitemia. Cultures were synchronized until >80% of parasites were in ring-stage growth and then treated for 1 h with 10 nM rap to excise *pfap2μ*. Cultures treated with dimethyl sulfoxide (DMSO) were used as negative controls. Parasites were then washed twice with RPMI and incubated at 37°C until harvesting. Synchronized parasites were magnetically sorted as either trophozoite or schizont stages (MACS LD separation column; Miltenyi Biotech, Germany), collected by centrifugation, and fixed in 2% formaldehyde–2.5% glutaraldehyde (Polysciences, Inc., Warrington, PA) in 100 mM cacodylate buffer (pH 7.2) for 1 h at room temperature. The samples were washed in cacodylate buffer and postfixed in 1% osmium tetroxide (Polysciences, Inc.) for 1 h. The samples were then rinsed extensively in dH_2_O prior to *en bloc* staining with 1% aqueous uranyl acetate (Ted Pella, Inc., Redding, CA) for 1 h. After several rinses in dH_2_O, the samples were dehydrated in a graded series of ethanol-water mixes and embedded in Eponate 12 resin (Ted Pella, Inc.). Sections (90 nm) were cut with a Leica Ultracut UCT ultramicrotome (Leica Microsystems, Inc., Bannockburn, IL), stained with uranyl acetate and lead citrate, and viewed on a JEOL 1200 EX transmission electron microscope (TEM; JEOL USA, Inc., Peabody, MA) equipped with an AMT 8 megapixel digital camera and AMT Image Capture Engine V602 software (Advanced Microscopy Techniques, Woburn, MA).

### Immunoelectron microscopy.

Parasites at 2% hematocrit and 6 to 8% parasitemia were magnetically sorted from uninfected RBCs and ring-stage parasites as above, collected by centrifugation and fixed for 1 h at 4°C in 4% formaldehyde (Polysciences, Inc., Warrington, PA) in 100 mM PIPES–0.5 mM MgCl_2_ (pH 7.2). Samples were then embedded in 10% gelatin, infiltrated overnight with 2.3 M sucrose–20% polyvinyl pyrrolidone in PIPES-MgCl_2_ at 4°C, and finally trimmed, frozen in liquid nitrogen, and sectioned with a Leica Ultracut UCT7 cryo-ultramicrotome (Leica Microsystems). Next, 50-nm sections were blocked with 5% fetal bovine serum–5% normal goat serum (NGS) for 30 min, followed by incubation with a primary antibody for 1 h at room temperature (anti-PDI mouse, 1:100 [1D3; Enzo Life Sciences]; anti-GFP rabbit, 1:200 [A-11122; Life Technologies]; anti-GFP mouse, 1:100 [11814460001; Roche], anti-HA rabbit, 1:50 [H6908; Sigma-Aldrich]; and anti-Rab5A rabbit, 1:50, and anti-Rab5B rat, 1:50 [Gordon Langsley]). Secondary antibodies were added at 1:30 for 1 h at room temperature [12-nm Colloidal Gold AffiniPure goat anti-rabbit IgG(H+L) (111-205-144), 18-nm Colloidal Gold AffiniPure goat anti-rabbit IgG(H+L) (111-215-144), 12-nm Colloidal Gold AffiniPure goat anti-mouse IgG(H+L) (115-205-146), and 18-nm Colloidal Gold AffiniPure goat anti-mouse IgG+IgM(H+L) (115-215-068) (all from Jackson ImmunoResearch)]. Sections were then stained with 0.3% uranyl acetate–2% methyl cellulose and viewed on the JEOL TEM as described above. All labeling experiments were conducted in parallel with controls omitting the primary antibody; these were consistently negative under the conditions used in these studies.

### Antibodies.

Anti-HA (Roche, 3F10 clone) was obtained from the manufacturer. Anti-CHC (rabbit) antibodies were donated by Frances Brodsky. We thank Gordon Langsley for making the anti-Rab5B antibodies available. Anti-CDC48 and anti-AMA1 and anti-MSP1 antibodies were generously provided by Jude Przyborski and Michael Blackman, respectively. Anti-GFP, anti-GAP45, and anti-BiP were supplied by Anthony Holder. Anti-ERD2 was obtained from the MR4 repository. Secondary antibodies were either highly crossed adsorbed fluorophore conjugated (Invitrogen) or enzyme conjugated (Sigma). Primary and secondary antibodies were used at 1:150 and 1:250, respectively, for IFA and at 1:5,000 and 1:5,000, respectively, for Western blot experiments. Anti-CHC antibody was donated by Frances Brodsky and used at 1:500 for IFA and at 1:20,000 for Western blotting. All antibodies were generously donated by other laboratories and had been raised against P. falciparum antigens with validation against lysates of cultured parasites. Cross-reactivity with other *Plasmodium* species may occur with these reagents.

### Pulldown and mass spectrometry.

For lysate preparation, at 32 to 35 h postinvasion P. falciparum-infected erythrocytes were grown to approximately 8% parasitemia at 5% hematocrit in approximately 6 liters of complete medium, sedimented, and lysed with 0.15% (wt/vol) saponin in PBS at 4°C. Parasites were harvested by centrifugation at 13,000 rpm for 5 min at 4°C and then washed several times with cold PBS to remove hemoglobin and red cell debris. The washed, packed parasites were resuspended to 50% density in PBS, flash frozen in liquid nitrogen, and stored at –80°C. This process was repeated until 6 to 8 ml of resuspended parasites had been stored. The frozen material was placed directly into the ball chamber of a liquid N_2_-cooled cryomill (Retsch) and milled under seven cycles of 3 min of cooling and 3 min of milling. The milled powder was removed from the ball chamber and stored in liquid N_2_. All steps were performed at or below –80°C to prevent parasite material from thawing. A 300-mg portion of the milled powder per replicate was lysed in buffer A (20 mM HEPES, 100 mM NaCl, 0.1% [vol/vol] Triton X-100, 0.1 mM TLCK [*N*α-*p*-tosyl-l-lysine chloromethyl ketone], and protease inhibitors [Complete protease inhibitor cocktail tablet, EDTA-free, Roche]; pH 7.4) or buffer B (20 mM HEPES, 250 mM sodium citrate, 0.1% [wt/vol] CHAPS, 1 mM MgCl_2_, 10 mM CaCl_2_, 0.1 mM TLCK, and protease inhibitors; pH 7.4). Buffer B was previously optimized to immunoprecipitate clathrin heavy chain from trypanosomes (45). The lysate was sonicated on ice with four cycles of 3 s on at 30% amplitude, followed by 10 s off and clarified by centrifugation. For 3D7-AP-2μ-3xHA, the soluble extract was incubated with 240 μl of anti-HA magnetic beads (Pierce, Thermo Fisher) for 1 h. The beads were washed three times with lysis buffer, and bound material was eluted by suspending the beads in 80 μl of nonreducing LDS buffer (Invitrogen) and incubating at 70°C for 10 min. After the beads were removed, NuPAGE sample-reducing agent (Thermo Fisher) was added to the supernatant.

The PfCHC-2xFKBP-GFP soluble extract in buffer B was incubated with 4 μl of recombinant anti-GFP nanobodies covalently coupled to surface-activated Epoxy magnetic beads (Dynabeads M270 Epoxy, Thermo Fisher) for 1 h, washed three times in buffer B and eluted in 80 μl of LDS buffer (Invitrogen), supplemented with NuPAGE, at 70°C for 10 min. The eluates were concentrated in a Speed-Vac to 30 μl and run approximately 1.2 cm into an SDS-PAGE gel. The respective gel region was sliced out and subjected to tryptic digest, reductive alkylation. Eluted peptides were analyzed by liquid chromatography-tandem mass spectrometry on a Dionex UltiMate 3000 RSLCnano System (Thermo Scientific, Waltham, MA) coupled to an Orbitrap Q Exactive mass spectrometer (Thermo Scientific) at the University of Dundee Finger-Prints Proteomics facility. Mass spectra were processed using MaxQuant version 1.5 by the intensity-based label-free quantification (LFQ) method (59, 60). The minimum peptide length was set at six amino acids, and false discovery rates of 0.01 were calculated at the levels of peptides, proteins, and modification sites based on the number of hits against the reversed sequence database. Ratios were calculated from LFQ intensities using only peptides that could be uniquely mapped to a given protein across two (AP-2μ CHAPS) or four (AP-2μ Triton, CHC CHAPS) replicates of each treatment/bait protein combination. The software Perseus was used for statistical analysis of the LFQ data (60). Extended proteomic data tables are available at https://doi.org/10.17037/DATA.00001533.

10.1128/mBio.02918-19.10 TABLE S2 Primers used to screen transgenic lines described in this study. Download Table S2, DOCX file, 0.1 MB. Copyright © 2020 Henrici et al. 2020 Henrici et al. This content is distributed under the terms of the Creative Commons Attribution 4.0 International license.
