# Supplementary material for: The Plasmodium falciparum Artemisinin Susceptibility-Associated AP-2 Adaptin μ Subunit is Clathrin Independent and Essential for Schizont Maturation
Source: mBio. 2020 Feb 25;11(1):e02918-19. doi: 10.1128/mBio.02918-19 (PMC7042695; doi:10.1128/mBio.02918-19)
Supplement: TABLE S1 [file mBio.02918-19-st001.docx]

| **Localisation to structure (% of cells)** | **AP-2μ-3xHA** | **AP-2μ-GFP** |
| --- | --- | --- |
| Tubular ER extension / Golgi | 93.6% | 87.6% |
| Vesicles | 37.9% | 30.0% |
| Cytoplasm (non-membranous) | 7.6% | 8.0% |
| Digestive vacuole | 5.8% | 4.3% |
| Periphery / parasite plasma membrane | 4.2% | 3.9% |

**Suppl. Table 1. Quantitation of distribution of gold labels in immunoelectron micrographs.**

Micrographic images of 66 erythrocytes infected with trophozoite stage parasites expressing 3xHA-tagged AP-2μ, stained with gold particles conjugated to anti-HA antibodies, were examined. For comparison, a similar number of cells infected with trophozoites expressing AP-2µ-2xFKBP-GFP,^32^ stained with gold particles conjugated to anti-GFP antibodies, were examined.
