# Supplementary figures and images for: The Plasmodium falciparum Artemisinin Susceptibility-Associated AP-2 Adaptin μ Subunit is Clathrin Independent and Essential for Schizont Maturation
Source: mBio. 2020 Feb 25;11(1):e02918-19. doi: 10.1128/mBio.02918-19 (PMC7042695; doi:10.1128/mBio.02918-19)

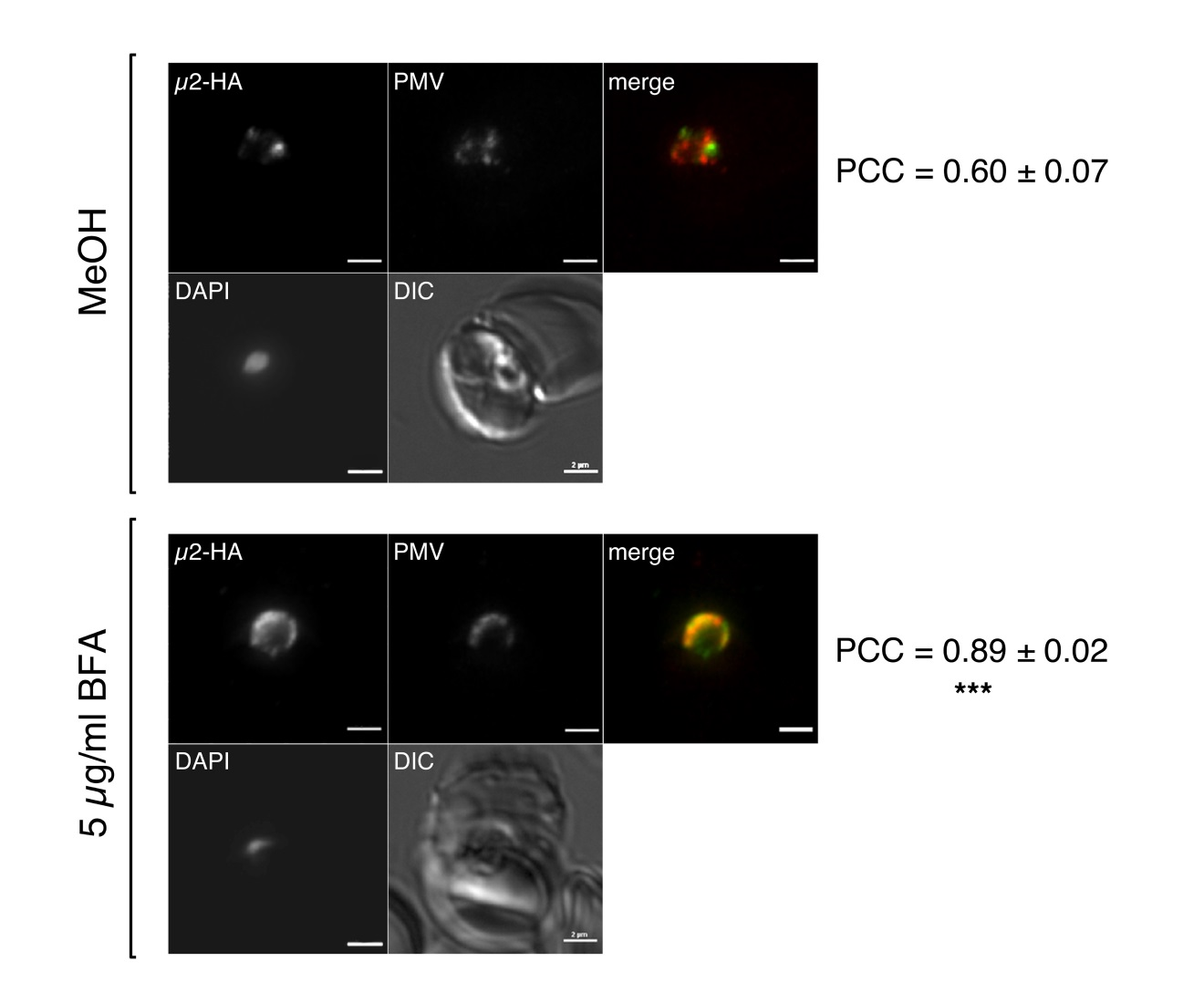

Supplement: FIG S4 [file mBio.02918-19-sf004.tif]
