# Supplementary material for: The Plasmodium falciparum Artemisinin Susceptibility-Associated AP-2 Adaptin μ Subunit is Clathrin Independent and Essential for Schizont Maturation
Source: mBio. 2020 Feb 25;11(1):e02918-19. doi: 10.1128/mBio.02918-19 (PMC7042695; doi:10.1128/mBio.02918-19)
Supplement: TABLE S2 [file mBio.02918-19-st002.docx]

**Suppl. Table 2. Primers used to screen transgenic lines described in this study**
